# A Clinical Lecture

**Published:** 1905-06

**Authors:** William Seaman Bainbridge

**Affiliations:** Surgeon New York Skin and Cancer Hospital; Attending Surgeon New York Children’s Hospitals and Schools; Adjunct Professor of Operaive Surgery and Operative Gynecology, New York Post-Graduate Medical School and Hospital; Consultant New York Home for Destitute Crippled Children


### A CLINICAL LECTURE.*

By WILLIAM SEAMAN BAINBRIDGE, A.M., M.D.,

Surgeon New York Skin and Cancer Hospital; Attending Surgeon New York Children's Hospitals and Schools; Adjunct Professor of Operaive Surgery and Operative Gynecology, New York Post-Graduate Medical School and Hospital; Consultant New York Home for Destitute Crippled Children.

A clinical lecture on cancer was recently delivered by Dr. William Seaman Bainbridge in the clinic-room of the New York Skin and Cancer Hospital. A number of patients were presented whose cases illustrate the different stages of cancerous disease and the results of operative treatment.

In his introductory remarks Dr. Bainbridge emphasized the fact that at no time in the history of America, and probably of the world at large, has the subject of cancer been so much in the minds of the people as at present, partly because there is apparently a real increase in its frequency, because it has carried off so many distinguished and useful citizens, and also be-

*Reported by Loy McAfee, Ingraham, M.D.

cause of the many theories as to its origin and cure which have been exploited within the last few years. He quoted statistics from the United States Census to show that from 1850 to 1890 the mortality from cancer increased from 9 to 33.5 to every 100,000 population. The more accurate diagnosis of the present time, and the fact that more cases are reported, do not entirely account for this statistical increase.

In speaking of the unsatisfactory status of our present knowledge of cancer etiology, Dr. Bainbridge dwelt upon certain practical facts which are so far established as to be accepted by the medical profession. The more important of these are as follows:

1. All cancer begins as a benign growth.

2. There is, therefore, a true precancerous stage, in which removal is a sure means of relief.

3. The disease is absolutely local in its beginning, and if fully extirpated a cure should result.

4. Extension may take place by direct infection of the surrounding tissue, but it is usually through the lymphatic or blood channels.

5. There is a varying degree of malignancy, some growths tending to return much more readily than others.

6. The system is poisoned by the production of toxins.

7. General malnutrition, as well as diminished vitality of the noncancerous tissue in the neighborhood of malignant disease, as a rule, tends to increase the rapidity of the local extension and renders more likely the development of metastases.

The treatment of cancer was briefly epitomized by Dr. Bainbridge as follows:

I. *Non-operative Treatment.*—(1) Arsenic paste and other caustics; (2) liquid air, X-ray, Finsen light and radium; (3) bacteriotherapy; (4) serum therapy, which is, as yet, only in its experimental stage.

II. *Operative Treatment.*—(1) Removal, as far as possible, of all benign growths, especially those subject to chronic irritation or repeated trauma; (2) when malignancy is present, early removal of the cancer, with a margin of a quarter or a half inch of healthy tissue, and the extirpation of lymphatic vessels and lymphatic nodes in close relation to the disease; (3) in advanced cases as radical operation as is compatible with life; (4) when it is impossible to remove the disease, palliative operation for the amelioration of suffering and the prolongation of life.

The cases presented were classified as follows: 1. Precancerous stage (benign tumors). 2. Early cases. 3. Advanced cases. 4. Cases of irremovable but operable cancer.

The following patients were presented to illustrate the first class:

1.—*Angioma Hypertrophicum,* in a boy, about two years of age. Child was perfect at birth, but when two weeks old a small blue spot appeared under the skin of the upper lip, just below the septum of the nose. This increased in size rapidly until at the time of operation, May 6, 1904, the tumor practically embraced all the upper lip, extending well up around the alæ nasi and somewhat into the nasal cavity. The growth was extirpated as completely as possible, the incision extending on to the septum in both nostrils and around the alæ nasi. To prevent sloughing from too great tension a considerable redundancy of mucous membrane was left at the vermilion border. This redundancy was removed five months later and the lip shaped. When presented at the clinic, five months after the second operation, the scar is practically imperceptible. No tendency to return of growth.

2.—*Periconicular Fibroma of Breast,* in a single woman twenty years of age. At the time of operation, March 15, 1905, the entire right breast was found to be somewhat enlarged, nipple not retracted; a tumor the size of a lemon was found below the nipple, and two smaller tumors on either side of the breast, near the margin. Several enlarged glands found in the axilla. Through a circular incision below and to the outer side of the breast, at the juncture of the breast mass and the chest wall, the breast was separated from the pectoralis major and the capsule incised. Through incisions radiating toward the nipple in the substance of the gland the tumors were excised. The enlarged axillary glands were removed through the first incision.

The capsule of the gland was sutured with catgut, the entire mamma replaced, and the wound closed, absolutely no mutilation resulting.

3.—*Fibrolipoma of Back,* resulting from a sharp blow received two years previous to appearance of growth, and five years before operation. Female, colored, aged forty-four. At the time of operation, May 18, 1904, tumor weighed three pounds and was found to develop from the deep connective tissue between the first and second layers of muscles, two and one-half inches below the scapula. Removed by circular incision over the most prominent part of the tumor. Wound healed by primary union and patient was discharged in eight days. Very little pseudo-keloid formation, which, as well as true keloid, is so common among negroes, resulted.

A number of patients were presented to illustrate the second class, early cases of cancer, possibly the most interesting being the following:

1.—*Carcinoma of Right Breast,* in a woman seventy-three years of age. When operated upon, November 21, 1904, the tumor was about the size of a small egg, hard, nodular, adherent to the skin, but movable on the pectoral muscles. For about three weeks before operation tumor was tender, and pain was referred to axilla. A modified Halsted operation was done, the entire breast, the larger part of the pectoralis major, and the glands of the axilla being removed. The patient demonstrated to those present that she had perfect use of the arm on the affected side.

2.—*Epithelioma of Lower Lip and Glands of Neck.* Male, age 75. About a year ago a small pimple appeared on the left side of the lower lip, disappearing in about three months under treatment with a caustic paste. Five months later a similar pimple appeared on the right side of the lip, but this persisted despite caustic pastes and electricity. The patient came under Dr. Bainbridge's care early in December, 1904. At the time of operation, December 29, the glands of the neck were not palpable, there was a hard mass on the right side of the lip and a slight indurated spot on the left side remaining after the use of caustic paste. Under chloroform and oxygen vapor anesthesia a wedge-shaped piece was taken from the right side of the lip, also a small wedge-shaped piece from the scar on the left side. The submaxillary gland and others in close relation therewith were removed from the right side. The wound was closed with silk and healed by primary union. The apparently unaffected glands on the left side were left pending pathological report. This report showed squamous-cell epithelium on the right side of the lip and glands of neck; left side negative. Acting upon this pathological diagnosis the patient was discharged. Two months later he returned to the hospital with palpable glands in the left side of the neck. Recurrence probably came from the left side, which, despite the negative pathological report, was undoubtedly cancerous. At a second operation, March 27, 1905, all glands in the left side of the neck were removed, and a suspicious spot of hardness on the right side at the base, close to the alveolar process, was excised.

The third class, advanced cases, was illustrated by several patients, the two most interesting cases being given below.

1.—*Epithelioma of Nose.* Female, aged 60. For thirteen years has been treated in various cities and by various methods, but despite all interference the cancer has steadily grown worse. Operation February 15, 1905. An incision was made over the middle line of the nose, under the eyebrow, under the eye, down on the cheek. All diseased tissue, including a portion of the nasal process of the superior maxilla, was removed, the eye and the nasal duct being saved. A month later the edges of the wound were freshened and the surface of the wound covered by a flap taken from the inner surface of the arm on the affected side. The arm was placed over the head, the flap, still attached to the arm, was turned over and sutured by its inner margin to the face, and the head and arm encased in plaster of paris, the opposite side of the face being exposed. When the wound was dressed five days after the operation the flap was apparently healthy.*

2.—*Extensive Carcinoma of Tongue and Neck.* Male, aged 49. Mother died of cancer of breast at age of 74. No history of spe-

*The flap has been detached and at the present time the wound is entirely healed.

cific disease. Used alcohol freely. Smoked twenty cigars a day for twenty years. Cigar always held in left side of mouth, resting against that part of the tongue which subsequently became the seat of the disease. In May, 1902, patient first noticed a small pimple, half-way back on the dorsum of the tongue. When he came under Dr. Bainbridge's observation (December 26, 1903) there was a hard, crater-like ulcer (3 by 2 by 4.5 cm.), involving the left anterior third of the tongue, except at the tip. No glands palpable at this time. Microscopic examination of a section of the ulcer, made by two independent authorities, confirmed the diagnosis of a very vascular epithelioma. Immediate operation being refused, potassium iodide, a mouth wash and tonics were prescribed, and systematic X-ray treatment employed for nine weeks. The patient's general and local condition grew steadily worse. This treatment was discontinued March 1, by which time the growth had extended to the right half of the tongue and induration of the floor of the mouth was evident. Some cervical glands were distinctly palpable on both sides of the neck.

On March 11, under chloroform and oxygen vapor anesthesia, the following operation was performed: First incision, across the neck practically from the tip of the left mastoid process to that of the right, and below as far as the thyroid cartilage. Second incision, along the anterior border of the left sterno-cleido-mastoid muscle to within an inch of the clavicle. The submaxillary and sublingual glands on either side were removed and the salivary ducts extirpated clear into the mouth. Many cancerous glands were removed from the region of the tonsil on the left side to the dome of the pleura, and on the right side from the tonsil to the division of the carotid artery. The wound was closed except for a small drain at the lower part. The mouth was next forced open, part of the large cauliflower mass on the tongue cut down with Paquelin cautery. The cauterized surface was coated daily with Whitehead's shellac. The wound healed by primary union.

On March 28th a second operation was performed, when the left corner of the mouth was incised as far back as the edge of the masseter muscle, the tongue drawn out and completely re-removed by an elliptical incision on the floor of the mouth encircling the tongue in front and on each side. A flap of mucous membrane and muscle from the right glosso-epiglottic fold was used in making a bridge of tissue across the fauces in front of the epiglottis. The wound in the floor of the mouth was closed by chromicized catgut and covered with shellac. The wound in the cheek was closed in the usual way and shellac applied.

April 21, the patient was discharged cured. He weighed on entering the hospital 129 pounds, 139 when discharged, and 160 to-day. He is apparently perfectly well, is able to masticate even solid food, to taste, to talk intelligently, even to sing.

Illustrating the fourth class, the irremovable, but operable cases, the following patients were presented:

1.—*Epithelioma of Tongue.* Male, thirty-six years of age. For three years was treated for syphilis, which was not present. When epithelioma was recognized the right half of the tongue was involved by an ulcerated tumor the size of a quarter, a large number of the glands of the neck on the affected side were involved, the patient was suffering intense pain, and the condition had progressed to such an extent that there was little chance of total removal of the disease. November 25, 1904, the glands of the neck and the right half of the tongue were removed. In March, 1905, the patent returned to the hospital. He had been apparently well up to three weeks before, when the neck began to swell just below the ear and he suffered considerable pain. A second operation was performed, when the external carotid artery on one side was extirpated in its entirety, by the Dawbarn method, each branch being tied off separately.* As much as possible of the diseased tissue was removed. No recurrence found in the mouth.

2.—*Carcinosis of Abdominal Organs.* Male, orthodox Hebrew, forty years of age. Had been treated for a year at eight or ten dispensaries for indigestion. When he entered the hospital, the latter part of December, 1903, he was vomiting everything, and was very weak and emaciated. Stimulants and rectal feeding had to be resorted to. There was tenderness in the region of the stomach, where a tumor the size of an orange could be felt. When

* Three weeks after second operation the external carotid artery on the opposite side was extirpated by the Dawbarn method. Patient has been discharged much improved operated, January 5, 1904, a cancerous growth was found to involve part of the greater curvature and posterior wall of the stomach, with some constriction at the pylorus. There were nodules of carcinoma on the liver, in the omentum and transverse mesocolon. An anterior gastro-jejunostomy was performed by the McGraw method. The patient made an uninterrupted recovery, and at the end of ten weeks, resumed his work as a tailor.

In May, 1904, he began to vomit again. In this instance, however, vomiting differed from the first, in that he would eat all day, his abdomen would swell, he would retch, but could not relieve himself by vomiting, until he pressed his hands over the abdomen from right to left.

The patient was operated upon a second time in June, 1904, when the growth was found to have invaded the artificial communication between the stomach and the intestine, shutting off completely the passage of food into the distal loop of the intestine; thus the proximal arm of the gut was dilated, forming, as it were, an accessory stomach in which the food accumulated during the day. Another communication was established between the stomach and intestine by the McGraw method between the dilated proximal arm of gut and the anterior wall of the stomach high up, and between the proximal intestinal arm and the intestine below. The patient made a speedy recovery, and six weeks after this operation began doing full work as a tailor. During the last three months he has had considerable pain, and the mass in the abdomen has grown steadily in size. He has lost a little flesh lately, but still weighs 135 pounds.

